# Supplementary material for: Swarm learning with weak supervision enables automatic breast cancer detection in magnetic resonance imaging
Source: Commun Med (Lond). 2025 Feb 6;5:38. doi: 10.1038/s43856-024-00722-5 (PMC11802753; doi:10.1038/s43856-024-00722-5)
Supplement: Supplementary file 2 — Supplemenntary information [file 43856_2024_722_MOESM2_ESM.pdf]

Supplementary Information

Title: Swarm learning with weak supervision enables automatic breast cancer detection in magnetic resonance imaging

Oliver Lester Saldanha (1, 2,*), Jiefu Zhu (1,*), Gustav Müller-Franzes (2), Zunamys I. Carrero (2), Nicholas R. Payne (3), Lorena Escudero Sánchez (3,4), Paul Christophe Varoutas (5), Sreenath Kyathanahally (6,7), Narmin Ghaffari Laleh (1), Kevin Pfeiffer (1), Marta Ligero (1), Jakob Behner (1), Kamarul A. Abdullah (3,8), Georgios Apostolakos (5), Chrysafoula Kolofousi (5), Antri Kleanthous (5), Michail Kalogeropoulos (5), Cristina Rossi (6,7), Sylwia Nowakowska (6), Alexandra Athanasiou (5), Raquel Perez-Lopez (9), Ritse Mann (10, 11), Wouter Veldhuis (12), Julia Camps (13), Volkmar Schulz (14,15), Markus Wenzel (14,16), Sergey Morozov (17), Alexander Ciritsis (6), Christiane Kuhl (2),
Fiona J. Gilbert (3), Daniel Truhn (2,*), Jakob Nikolas Kather (1,18,19,*,†)

(1) Else Kroener Fresenius Center for Digital Health, Medical Faculty Carl Gustav Carus, Technical University Dresden, Dresden, Germany.
(2) Department of Diagnostic and Interventional Radiology, University Hospital RWTH Aachen, Aachen, Germany.
(3) Department of Radiology, Clinical School, Cambridge Biomedical Research Centre, University of Cambridge, Cambridge, CB2 0QQ, UK.
(4) Cancer Research UK Cambridge Centre, Li Ka Shing Centre, Cambridge CB2 0RE, UK.
(5) Breast Imaging Department, Mitera Hospital Athens, Greece.
(6) Institute of Diagnostic and Interventional Radiology, University Hospital Zurich, Switzerland.
(7) b-rayZ AG, Wagistrasse 21, 8952, Schlieren, Switzerland.
(8) Universiti Sultan Zainal Abidin, Kampus Gong Badak, 21300 Kuala Nerus, Terengganu, Malaysia.
(9) Radiomics Group, Vall d'Hebron Institute of Oncology (VHIO), Barcelona, Spain.
(10) Department of Diagnostic Imaging, Radboud University Medical Center, Nijmegen, Netherlands.
(11) Department of radiology, the Netherlands Cancer Institute, Amsterdam, Netherlands.
(12) Imaging Division, University Medical Center Utrecht, Utrecht, Netherlands.
(13) Breast Cancer Unit, Ribera Salud Hospitals, Valencia, Spain.
(14) Fraunhofer Institute for Digital Medicine MEVIS, Bremen, Germany.
(15) Physics of Molecular Imaging, Experimental Molecular Imaging, RWTH Aachen University, Aachen, Germany.
(16) Constructor University Bremen GmbH, Bremen, Germany
(17) The European Society of Medical Imaging Informatics (EuSoMII), Vienna, Austria
(18) Medical Oncology, National Center for Tumor Diseases (NCT), University Hospital Heidelberg, Heidelberg, Germany.
(19) Department of Medicine 1, University Hospital and Faculty of Medicine Carl Gustav Carus, Technische Universität Dresden, Dresden, Germany.

(*) Contributed equally
(†) Corresponding Author

Supplementary Methods

Model Comparison Based on Technical Characteristics

The best model for a specified task is influenced by multiple factors, including its accuracy, generalizability, and efficacy. 3D-ResNet101 consistently showed high performance in both internal and external validation experiments for both SL and centralized model techniques, as we showed in previous sections. According to our results, the number of parameters in this 3D-ResNet101 model is 123.65 million, which is almost four times more than the 33.16 million parameters in 3D-ResNet18. Therefore, training this model is more computationally expensive than training the other models, although more efficient at the same time (262 minutes for 50 epochs of SL training, calculating 123.65 million model parameters for 3D-ResNet101, versus 127 minutes for 50 epochs of SL training, calculating 33.16 million model parameters for 3D-ResNet18). All the model architectures within this study are listed in (Suppl. Table 12), along with the training hyperparameters, the number of learnable parameters, and the size and the time required for training.

Exploring the Explainability of the Models

Lastly, we sought to verify the explainability of the models' predictions. Given the propensity of deep learning systems for overfitting and focusing on data artifacts and biases, especially when provided only with patient labels and no region annotations, we needed to ensure that the DL models had genuinely learned to identify the tumor and focus on the tumor in the slice. To this end, we employed various explainability approaches, including GradCAM, GradCAM++, and OCA. We found that GradCAM++ highlights image regions that are not relevant for the diagnosis (Fig. 3A-B). On the contrary, our results show that the OCA method focused only on malignant enhancing lesions (Suppl. Figure 7A-B). Overall, the explainability maps confirmed that the model focused on the tumor area in each image, but we found that OCA was more precise in locating the relevant image region and leaving irrelevant image regions out of consideration.

Supplementary Figures

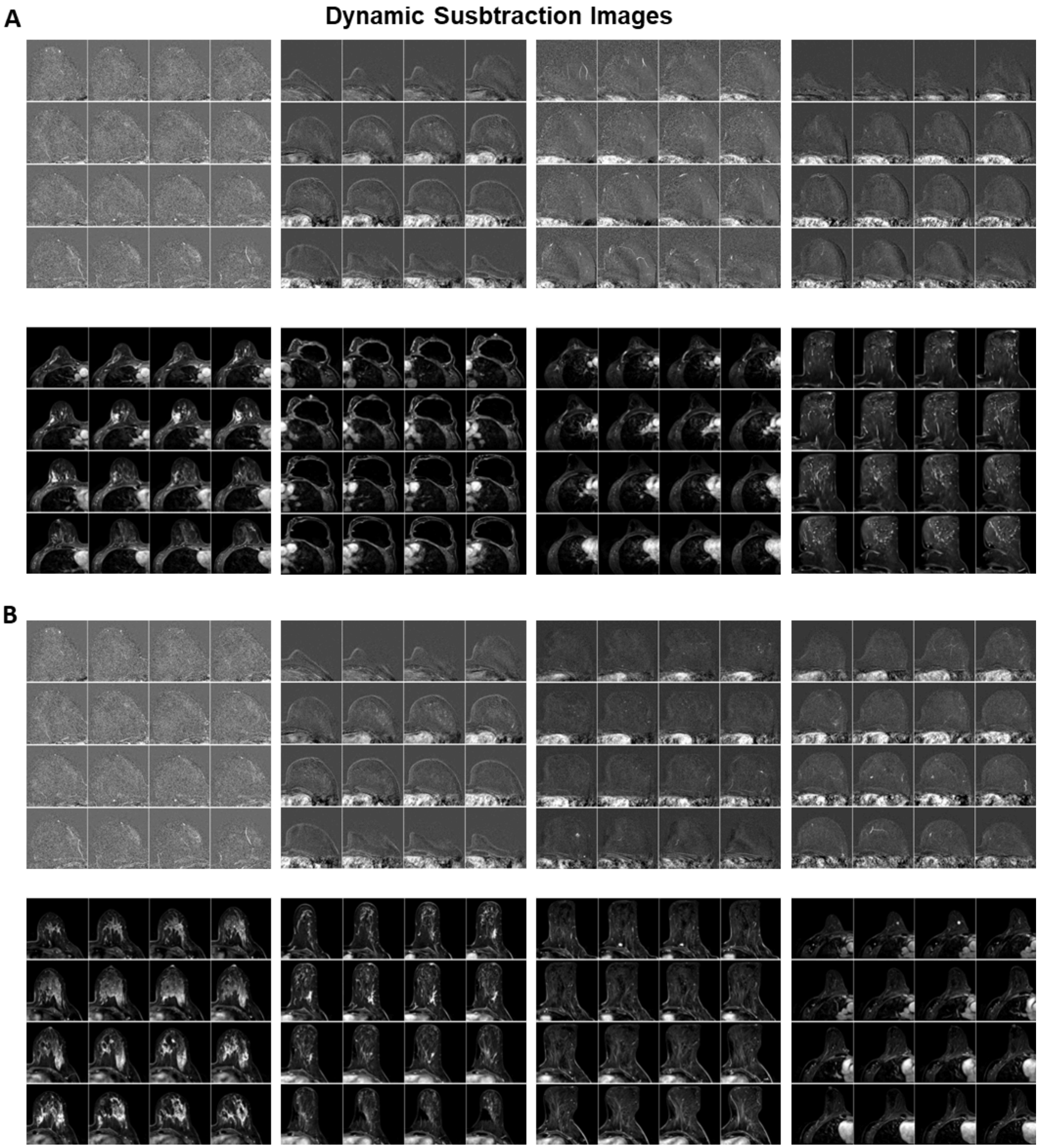

Suppl. Figure 1: Visual representation of the data and predictions on UKA and MHA external cohort on the 3D-ResNet-101 SL model trained on Duke, USZ, and CAM datasets. Each grid displays the 16 slices of a 3D volume, offering a comprehensive representation of the patient's data. **(A)** The first row displays the top four predicted true negative patients from the UKA cohort, while the second row illustrates the best four predicted true negative patients from

the MHA cohort. This category highlights instances where our model accurately identified negative outcomes with high confidence**. (B)** The first row displays the worst four predicted false negative cases from the UKA cohort, while the second row illustrates the worst four predicted false negative cases from the MHA cohort. This category highlights cases where our model incorrectly classified patients as negative, indicating areas for improvement.

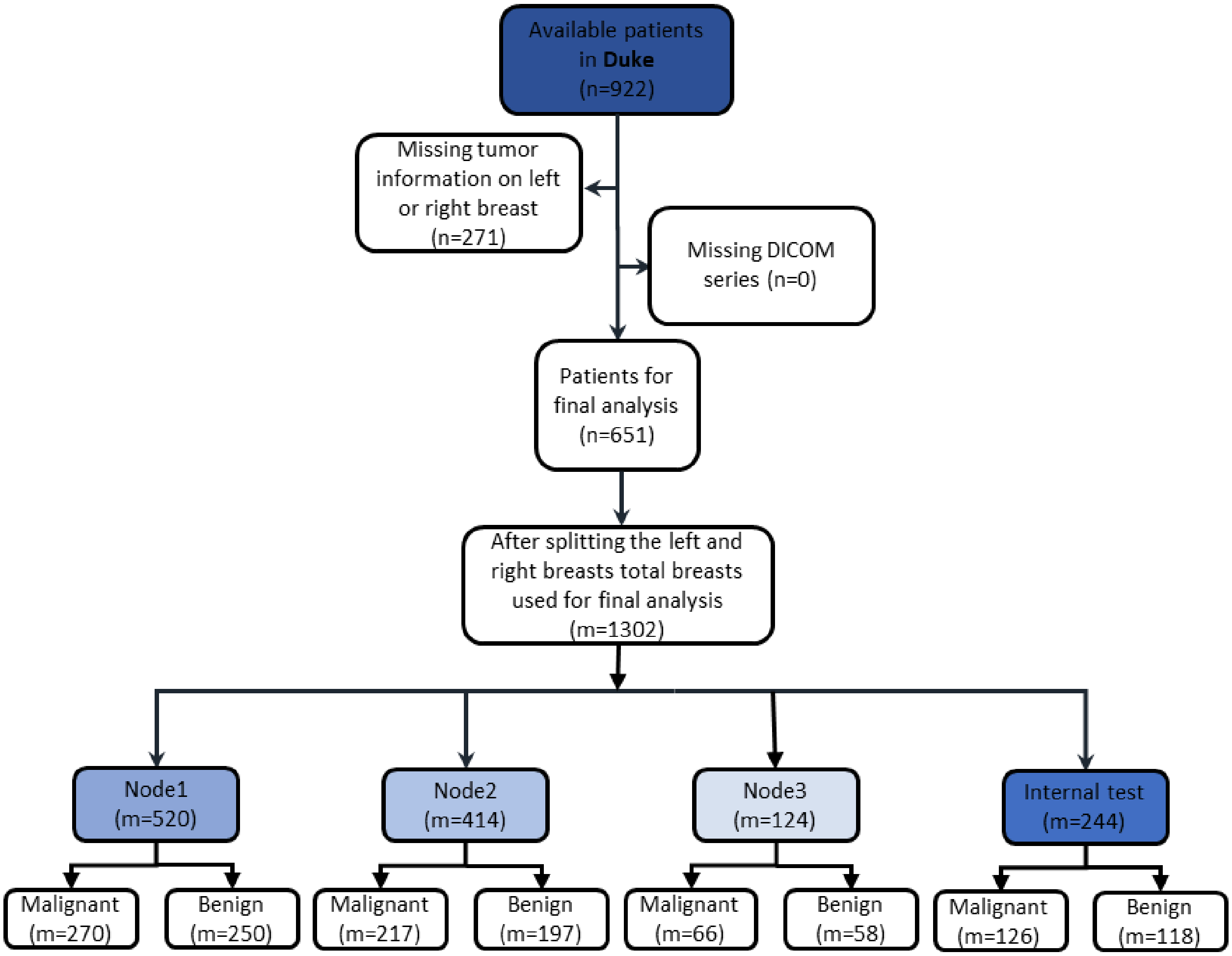

Suppl. Figure 2: Patient CONSORT diagram for the Duke cohort depicts the patient selection process."Missing tumor information on the left or right breast" signifies the absence of information if the tumor was in the right or left breast, while "Missing DICOM series" indicates the unavailability of DICOM data for certain patients. The diagram illustrates in different sections the count of patients (n) used for analysis and breast (m) after they are split into left and right and given the label malignant and benign based on the biopsy confirmed ground truth.

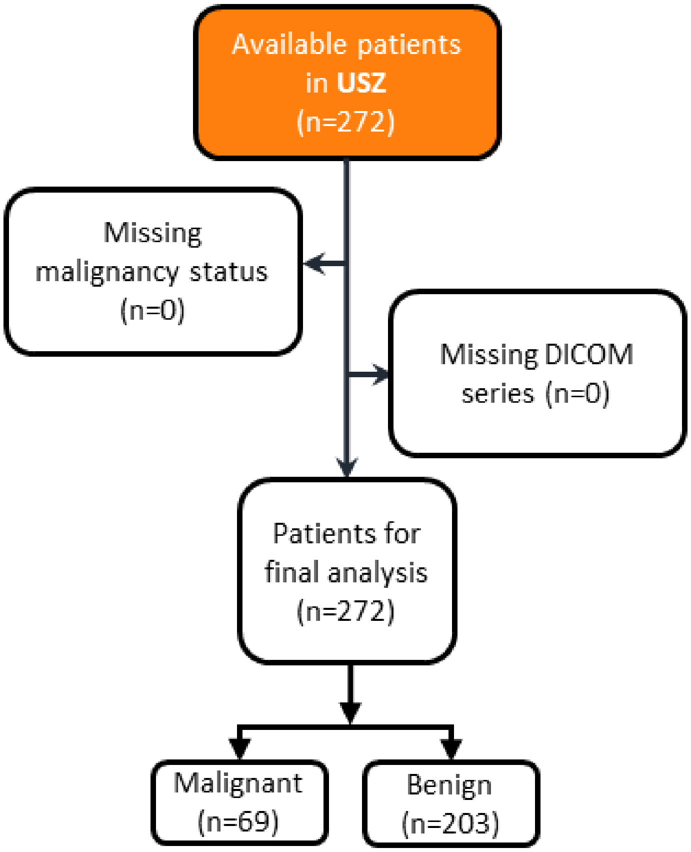

Suppl. Figure 3: Patient CONSORT diagram for the USZ cohort depicts the patient selection process."Missing malignancy status" signifies the absence of tumor status information, while "Missing DICOM series" indicates the unavailability of DICOM data for certain patients. The diagram illustrates the count of patients (n) used for analysis before they are split into different sections.

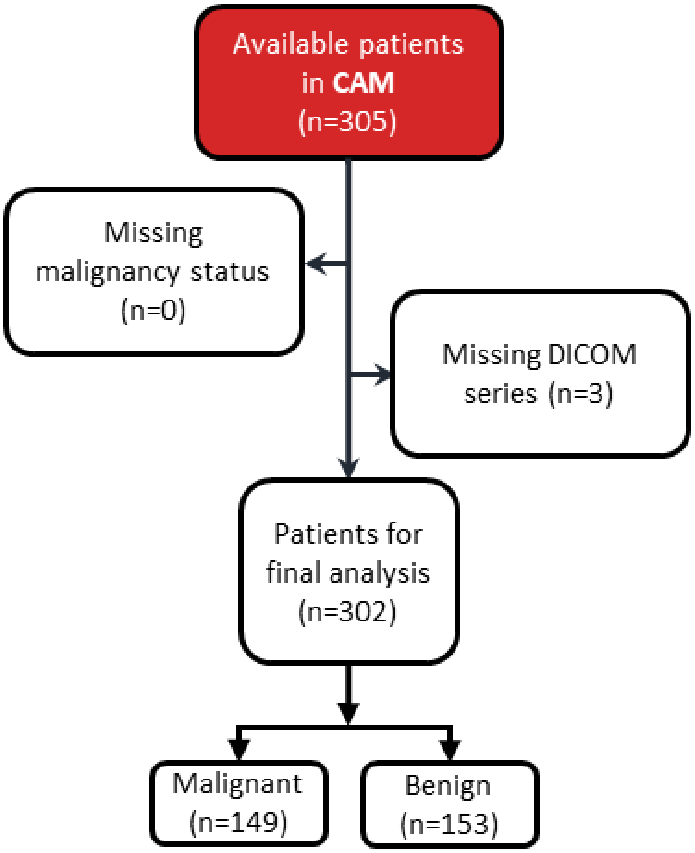

Suppl. Figure 4: Patient CONSORT diagram for the CAM cohort depicts the patient selection process."Missing malignancy status" signifies the absence of tumor status information, while "Missing DICOM series" indicates the unavailability of DICOM data for certain patients. The diagram illustrates the count of patients (n) used for analysis before they are split into different sections.

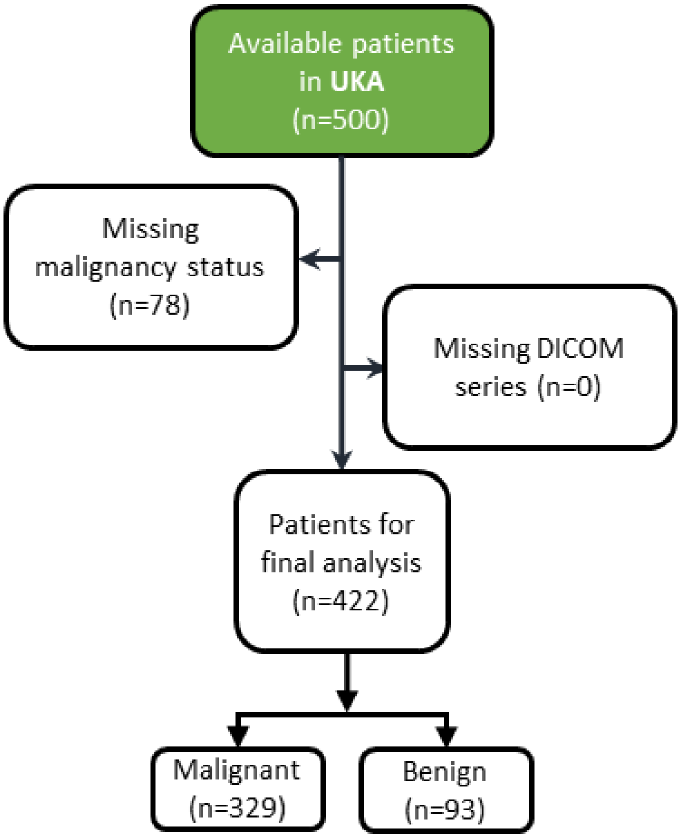

Suppl. Figure 5: Patient CONSORT diagram for the UKA cohort depicts the patient selection process. "Missing malignancy status" signifies the absence of tumor status information, while "Missing DICOM series" indicates the unavailability of DICOM data for certain patients. The diagram illustrates the count of patients used for analysis before they are split into different sections.

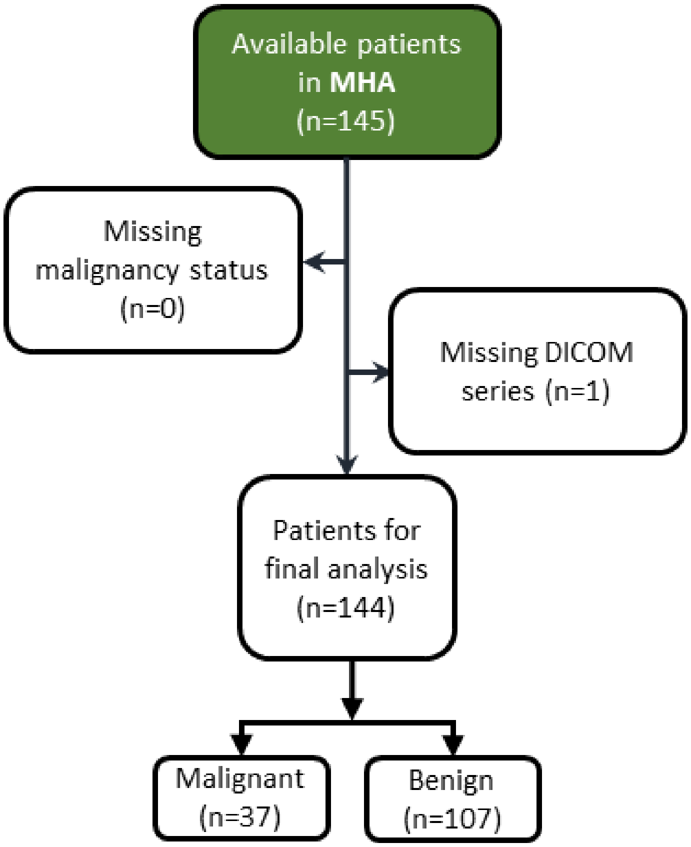

Suppl. Figure 6: Patient CONSORT diagram for the MHA cohort depicts the patient selection process. "Missing malignancy status" signifies the absence of tumor status information, while "Missing DICOM series" indicates the unavailability of DICOM data for certain patients. The diagram illustrates the count of patients used for analysis before they are split into different sections.

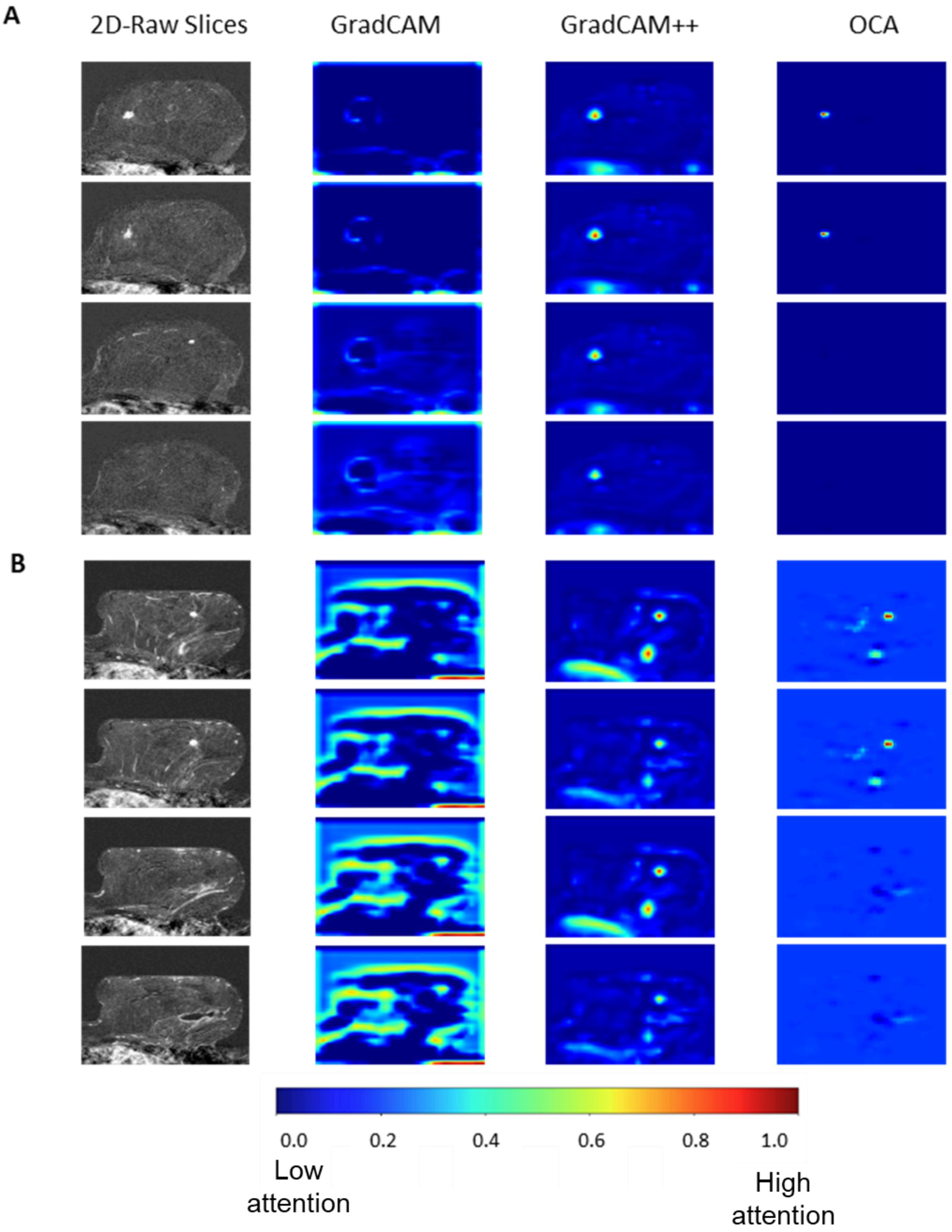

Suppl. Figure 7: Representative examples from UKA external test cohort for the explanatory maps when two lesions are present in the same breast trained on the real-world 3D-ResNet-101 SL model. A) invasive carcinoma (on the medial side of the breast) that is highlighted both by OCA and GradCAM++ but missed by GradCAM. All maps ignore the non-malignant adenosis in the ventrolateral quadrant. B) seroma (non-enhancing region, lateral)

and invasive carcinoma (enhancing 0,8x0,8cm region ventrolateral quadrant). The seroma is ignored by all maps, while the malignant carcinoma is picked up by GradCAM++ and OCA but not by GradCAM.

Supplementary Tables

	3D-ResNet 18	3D-ResNet 50	3D-ResNet 101	3D-DenseNet 121	ViT-MIL	ViT-LSTM-MIL	Att-MIL	3D-ResNet 50
**Techniques**	**Centralized Model**							
**3D-ResNet18**	1.000	0.337	0.086	**0.000**	0.484	0.299	**0.001**	**0.000**
**3D-ResNet50**	0.337	1.000	0.455	**0.000**	0.216	0.117	**0.000**	**0.000**
**3D-ResNet101**	0.086	0.455	1.000	**0.000**	0.104	0.05	**0.000**	**0.000**
**3D-DenseNet121**	**0.000**	**0.000**	**0.000**	1.000	**0.025**	0.057	0.649	0.051
**ViT-MIL**	0.484	0.216	0.104	**0.025**	1.000	0.732	**0.006**	**0.000**
**ViT-LSTM-MIL**	0.299	0.117	0.05	0.057	0.732	1.000	**0.017**	**0.000**
**Att-MIL**	**0.001**	**0.000**	**0.000**	0.649	**0.006**	**0.017**	1.000	0.143
**2D-ResNet50**	**0.000**	**0.000**	**0.000**	0.051	**0.000**	**0.000**	0.143	1.000
	**Swarm Learning**							
**3D-ResNet18**	1.000	**0.000**	0.054	0.524	0.648	0.571	0.091	**0.000**
**3D-ResNet50**	**0.000**	1.000	0.577	0.072	0.166	0.135	**0.009**	**0.000**
**3D-ResNet101**	0.054	0.577	1.000	**0.044**	0.096	0.075	**0.004**	**0.000**
**3D-DenseNet121**	0.524	0.072	**0.044**	1.000	0.932	0.982	0.257	**0.000**
**ViT-MIL**	0.648	0.166	0.096	0.932	1.000	0.912	0.215	**0.000**
**ViT-LSTM-MIL**	0.571	0.135	0.075	0.982	0.912	1.000	0.258	**0.000**
**Att-MIL**	0.091	**0.009**	**0.004**	0.257	0.215	0.258	1.000	**0.014**
**2D-ResNet50**	**0.000**	**0.000**	**0.000**	**0.000**	**0.000**	**0.000**	**0.014**	1.000

Suppl. Table 1: Significance comparison between models and benchmarking techniques for the internal cohort. We evaluated and compared models' performance based on AUROC using DeLong's test, considering a significance level of $p<0.05$ and median patient scores from five repetitions for determining better performance. All the p-values below the significance level are highlighted in bold.

Internal Validation<br>3D-ResNet18					
	**Node1<br>40% data**	**Node3<br>10% data**	**Node2<br>30% data**	**SL global best checkpoint**	**Centralized Model<br>80% data**
**Node1 - 40% data**	1.000	**0.000**	**0.000**	**0.000**	**0.000**
**Node3 - 10% data**	**0.000**	1.000	**0.000**	**0.000**	**0.000**
**Node2 - 30% data**	**0.000**	**0.000**	1.000	0.982	0.173
**SL global best checkpoint**	**0.000**	**0.000**	0.982	1.000	0.291
**Centralized Model<br>80% data**	**0.000**	**0.000**	0.173	0.291	1.000
**External Validation<br>3D-ResNet18**					
	**Node1<br>40% data**	**Node3<br>10% data**	**Node2<br>30% data**	**SL global best checkpoint**	**Centralized Model<br>80% data**
**Node1 - 40% data**	1.000	**0.001**	**0.000**	**0.002**	**0.000**
**Node3 - 10% data**	**0.001**	1.000	**0.000**	**0.000**	**0.000**
**Node2 - 30% data**	**0.000**	**0.000**	1.000	0.982	**0.000**
**SL global best checkpoint**	**0.002**	**0.000**	0.982	1.000	**0.000**
**Centralized Model<br>80% data**	**0.000**	**0.000**	**0.000**	**0.000**	1.000

Suppl. Table 2: Significance comparison between models for both internal and external test cohort. We evaluated and compared models' performance based on AUROC using DeLong's test, considering a significance level of $p<0.05$ and median patient scores from five repetitions for determining better performance. All the p-values below the significance level are highlighted in bold.

Internal Validation<br>3D-ResNet50					
	**Node1<br>40% data**	**Node3<br>10% data**	**Node2<br>30% data**	**SL global best checkpoint**	**Centralized Model<br>80% data**
**Node1 - 40% data**	1.000	**0.000**	0.073	0.213	**0.000**
**Node3 - 10% data**	**0.000**	1.000	**0.000**	**0.000**	**0.000**
**Node2 - 30% data**	0.073	**0.000**	1.000	0.714	0.873
**SL global best checkpoint**	0.213	**0.000**	0.714	1.000	0.556
**Centralized Model 80% data**	**0.000**	**0.000**	0.873	0.556	1.000
**External Validation<br>3D-ResNet50**					
	**Node1<br>40% data**	**Node3<br>10% data**	**Node2<br>30% data**	**SL global best checkpoint**	**Centralized Model<br>80% data**
**Node1 - 40% data**	1.000	**0.001**	0.073	0.213	**0.000**
**Node3 - 10% data**	**0.001**	1.000	**0.000**	**0.000**	0.117
**Node2 - 30% data**	0.073	**0.000**	1.000	0.714	**0.000**
**SL global best checkpoint**	0.213	**0.000**	0.714	1.000	**0.001**
**Centralized Model 80% data**	**0.000**	0.117	**0.000**	**0.001**	1.000

Suppl. Table 3: Significance comparison between models for both internal and external test cohorts. We evaluated and compared 3D-ResNet50 models performance based on AUROC using DeLong's test, considering a significance level of $p<0.05$ and median patient scores from five repetitions for determining better performance. All the p-values below the significance level are highlighted in bold.

Internal Validation<br>3D-ResNet101					
	**Node1<br>40% data**	**Node3<br>10% data**	**Node2<br>30% data**	**SL global best checkpoint**	**Centralized Model<br>80% data**
**Node1 - 40% data**	1.000	**0.000**	0.87	0.749	0.25
**Node3 - 10% data**	**0.000**	1.000	**0.000**	**0.000**	**0.000**
**Node2 - 30% data**	0.87	**0.000**	1.000	0.843	0.342
**SL global best checkpoint**	0.749	**0.000**	0.843	1.000	0.48
**Centralized Model<br>80% data**	0.25	**0.000**	0.342	0.48	1.000
**External Validation<br>3D-ResNet101**					
	**Node1<br>40% data**	**Node3<br>10% data**	**Node2<br>30% data**	**SL global best checkpoint**	**Centralized Model<br>80% data**
**Node1 - 40% data**	1.000	**0.000**	0.87	0.749	**0.019**
**Node3 - 10% data**	**0.000**	1.000	**0.000**	**0.000**	**0.000**
**Node2 - 30% data**	0.87	**0.000**	1.000	0.843	**0.013**
**SL global best checkpoint**	0.749	**0.000**	0.843	1.000	**0.008**
**Centralized Model<br>80% data**	**0.019**	**0.000**	**0.013**	**0.008**	1.000

Suppl. Table 4: Significance comparison between models for both internal and external test cohorts. We evaluated and compared 3D-ResNet101 models performance based on AUROC using DeLong's test, considering a significance level of $p<0.05$ and median patient scores from five repetitions for determining better performance. All the p-values below the significance level are highlighted in bold.

Internal Validation<br>3D-DensNet121					
	**Node1<br>40% data**	**Node3<br>10% data**	**Node2<br>30% data**	**SL global best checkpoint**	**Centralized Model<br>80% data**
**Node1 - 40% data**	1.000	0.457	**0.000**	0.871	0.921
**Node3 - 10% data**	0.457	1.000	**0.000**	**0.002**	0.377
**Node2 - 30% data**	**0.000**	**0.000**	1.000	**0.000**	**0.000**
**SL global best checkpoint**	0.871	**0.002**	**0.000**	1.000	0.956
**Centralized Model 80% data**	0.921	0.377	**0.000**	0.956	1.000
**External Validation<br>3D-DensNet121**					
	**Node1<br>40% data**	**Node3<br>10% data**	**Node2<br>30% data**	**SL global best checkpoint**	**Centralized Model<br>80% data**
**Node1 - 40% data**	1.000	0.212	**0.000**	0.458	0.456
**Node3 - 10% data**	0.212	1.000	**0.001**	0.371	**0.000**
**Node2 - 30% data**	**0.000**	**0.001**	1.000	**0.000**	**0.000**
**SL global best checkpoint**	0.458	0.371	**0.000**	1.000	0.102
**Centralized Model 80% data**	0.456	**0.000**	**0.000**	0.102	1.000

Suppl. Table 5: Significance comparison between models for both internal and external test cohorts. We evaluated and compared DesNet121-3D models performance based on AUROC using DeLong's test, considering a significance level of $p<0.05$ and median patient scores from five repetitions for determining better performance. All the p-values below the significance level are highlighted in bold.

Internal Validation<br>ViT-MIL					
	**Node1<br>40% data**	**Node3<br>10% data**	**Node2<br>30% data**	**SL global best checkpoint**	**Centralized Model<br>80% data**
**Node1 - 40% data**	1.000	**0.043**	0.787	0.106	**0.036**
**Node3 - 10% data**	**0.043**	1.000	**0.022**	**0.000**	**0.000**
**Node2 - 30% data**	0.787	**0.022**	1.000	0.178	0.068
**SL global best checkpoint**	0.106	**0.000**	0.178	1.000	0.632
**Centralized Model 80% data**	**0.036**	**0.000**	0.068	0.632	1.000

External Validation<br>ViT-MIL					
	**Node1<br>40% data**	**Node3<br>10% data**	**Node2<br>30% data**	**SL global best checkpoint**	**Centralized Model<br>80% data**
**Node1 - 40% data**	1.000	**0.036**	0.727	**0.019**	0.092
**Node3 - 10% data**	**0.036**	1.000	**0.015**	**0.000**	**0.000**
**Node2 - 30% data**	0.727	**0.015**	1.000	**0.049**	0.186
**SL global best checkpoint**	**0.019**	**0.000**	**0.049**	1.000	0.517
**Centralized Model 80% data**	0.092	**0.000**	0.186	0.517	1.000

Suppl. Table 6: Significance comparison between models for both internal and external test cohorts. We evaluated and compared ViT-MIL models performance based on AUROC using DeLong's test, considering a significance level of $p<0.05$ and median patient scores from five repetitions for determining better performance. All the p-values below the significance level are highlighted in bold.

Internal Validation<br>ViT-LSTM-MIL					
	**Node1<br>40% data**	**Node3<br>10% data**	**Node2<br>30% data**	**SL global best checkpoint**	**Centralized Model<br>80% data**
**Node1 - 40% data**	1.000	**0.000**	0.661	0.384	**0.000**
**Node3 - 10% data**	**0.000**	1.000	**0.000**	**0.000**	**0.000**
**Node2 - 30% data**	0.661	**0.000**	1.000	0.188	**0.000**
**SL global best checkpoint**	0.384	**0.000**	0.188	1.000	**0.000**
**Centralized Model<br>80% data**	**0.000**	**0.000**	**0.000**	**0.000**	1.000
**External Validation<br>ViT-LSTM-MIL**					
	**Node1<br>40% data**	**Node3<br>10% data**	**Node2<br>30% data**	**SL global best checkpoint**	**Centralized Model<br>80% data**
**Node1 - 40% data**	1.000	**0.000**	0.573	0.052	**0.007**
**Node3 - 10% data**	**0.000**	1.000	**0.007**	**0.000**	**0.000**
**Node2 - 30% data**	0.573	**0.007**	1.000	0.171	**0.036**
**SL global best checkpoint**	0.052	**0.000**	0.171	1.000	0.462
**Centralized Model<br>80% data**	**0.007**	**0.000**	**0.036**	0.462	1.000

Suppl. Table 7: Significance comparison between models for both internal and external test cohorts. We evaluated and compared ViT-LSTM-MIL models performance based on AUROC using DeLong's test, considering a significance level of $p<0.05$ and median patient scores from five repetitions for determining better performance. All the p-values below the significance level are highlighted in bold.

Internal Validation<br>Att-MIL					
	**Node1<br>40% data**	**Node3<br>10% data**	**Node2<br>30% data**	**SL global best checkpoint**	**Centralized Model<br>80% data**
**Node1 - 40% data**	1.000	**0.000**	**0.027**	0.458	**0.000**
**Node3 - 10% data**	**0.000**	1.000	**0.002**	**0.000**	**0.000**
**Node2 - 30% data**	**0.027**	**0.002**	1.000	0.144	**0.000**
**SL global best checkpoint**	0.458	**0.000**	0.144	1.000	**0.000**
**Centralized Model<br>80% data**	**0.000**	**0.000**	**0.000**	**0.000**	1.000
**External Validation<br>Att-MIL**					
	**Node1<br>40% data**	**Node3<br>10% data**	**Node2<br>30% data**	**SL global best checkpoint**	**Centralized Model<br>80% data**
**Node1 - 40% data**	1.000	**0.001**	**0.032**	0.967	**0.000**
**Node3 - 10% data**	**0.001**	1.000	0.257	**0.001**	0.757
**Node2 - 30% data**	**0.032**	0.257	1.000	**0.036**	0.14
**SL global best checkpoint**	0.967	**0.001**	**0.036**	1.000	**0.000**
**Centralized Model<br>80% data**	**0.000**	0.757	0.14	**0.000**	1.000

Suppl. Table 8: Significance comparison between models for both internal and external test cohorts. We evaluated and compared Att-MIL models performance based on AUROC using DeLong's test, considering a significance level of $p<0.05$ and median patient scores from five repetitions for determining better performance. All the p-values below the significance level are highlighted in bold.

Internal Validation<br>2D-ResNet50					
	**Node1<br>40% data**	**Node3<br>10% data**	**Node2<br>30% data**	**SL global best checkpoint**	**Centralized Model<br>80% data**
**Node1 - 40% data**	1.000	**0.000**	**0.024**	0.843	0.383
**Node3 - 10% data**	**0.000**	1.000	**0.000**	**0.000**	**0.000**
**Node2 - 30% data**	**0.024**	**0.000**	1.000	0.736	0.526
**SL global best checkpoint**	0.843	**0.000**	0.736	1.000	0.352
**Centralized Model 80% data**	0.383	**0.000**	0.526	0.352	1.000
**External Validation<br>2D-ResNet50**					
	**Node1<br>40% data**	**Node3<br>10% data**	**Node2<br>30% data**	**SL global best checkpoint**	**Centralized Model<br>80% data**
**Node1 - 40% data**	1.000	**0.000**	**0.014**	0.446	0.231
**Node3 - 10% data**	**0.000**	1.000	**0.000**	**0.000**	**0.000**
**Node2 - 30% data**	**0.014**	**0.000**	1.000	0.737	0.921
**SL global best checkpoint**	0.446	**0.000**	0.737	1.000	0.855
**Centralized Model 80% data**	0.231	**0.000**	0.921	0.855	1.000

Suppl. Table 9: Significance comparison between models for both internal and external test cohorts. We evaluated and compared ResNet50-2D model performance based on AUROC using DeLong's test, considering a significance level of $p<0.05$ and median patient scores from five repetitions for determining better performance. All the p-values below the significance level are highlighted in bold.

	**3D-ResNet 18**	**3D-ResNet 50**	**3D-ResNet 101**	**3D-DenseNet 121**	**ViT-MIL**	**ViT-LSTM-MIL**	**Att-MIL**	**3D-ResNet 50**
**Techniques**	**Centralized Model**							
**3D-ResNet18**	1.000	**0.000**	**0.000**	**0.018**	**0.013**	**0.003**	**0.001**	0.988
**3D-ResNet50**	**0.000**	1.000	**0.000**	0.115	0.194	0.43	**0.000**	**0.000**
**3D-ResNet101**	**0.000**	**0.000**	1.000	**0.000**	**0.003**	**0.013**	**0.000**	**0.000**
**3D-DenseNet121**	**0.018**	0.115	**0.000**	1.000	0.847	0.48	**0.000**	**0.018**
**ViT-MIL**	**0.013**	0.194	**0.003**	0.847	1.000	0.609	**0.000**	**0.013**
**ViT-LSTM-MIL**	**0.003**	0.43	**0.013**	0.48	0.609	1.000	**0.000**	**0.003**
**Att-MIL**	**0.001**	**0.000**	**0.000**	**0.000**	**0.000**	**0.000**	1.000	**0.000**
**2D-ResNet50**	0.988	**0.000**	**0.000**	**0.018**	**0.013**	**0.003**	**0.000**	1.000
	**Swarm Learning**							
**3D-ResNet18**	1.000	**0.000**	0.054	0.524	**0.007**	**0.001**	**0.000**	**0.000**
**3D-ResNet50**	**0.000**	1.000	0.577	0.072	**0.000**	**0.000**	**0.000**	**0.000**
**3D-ResNet101**	0.054	0.577	1.000	**0.044**	**0.000**	**0.000**	**0.000**	**0.000**
**3D-DenseNet121**	0.524	0.072	**0.044**	1.000	0.051	**0.010**	**0.000**	**0.000**
**ViT-MIL**	**0.007**	**0.000**	**0.000**	0.051	1.000	0.383	**0.002**	**0.000**
**ViT-LSTM-MIL**	**0.001**	**0.000**	**0.000**	**0.010**	0.383	1.000	**0.032**	**0.001**
**Att-MIL**	**0.000**	**0.000**	**0.000**	**0.000**	**0.002**	**0.032**	1.000	0.195
**2D-ResNet50**	**0.000**	**0.000**	**0.000**	**0.000**	**0.000**	**0.001**	0.195	1.000

Suppl. Table 10: Significance comparison for external cohort: Comparative analysis of benchmarking techniques using DeLong's test, considering a significance level of $p<0.05$ and

median patient scores from five repetitions. All the p-values below the significance level are highlighted in bold.

External Validation On UKA dataset<br>3D-ResNet101				
	**Duke**	**USZ**	**CAM**	**SL global best checkpoint**
**DUKE**	1.000	**0.000**	**0.012**	**0.035**
**USZ**	**0.000**	1.000	**0.000**	**0.000**
**CAM**	**0.012**	**0.000**	1.000	**0.000**
**SL global best checkpoint**	**0.035**	**0.000**	**0.000**	1.000
**External Validation on MHA<br>3D-ResNet101**				
	**Duke**	**USZ**	**CAM**	**SL global best checkpoint**
**DUKE**	1.000	**0.000**	**0.004**	**0.000**
**USZ**	**0.000**	1.000	**0.000**	**0.000**
**CAM**	**0.004**	**0.000**	1.000	**0.000**
**SL global best checkpoint**	**0.000**	**0.000**	**0.000**	1.000

Suppl. Table 11: Significance comparison between real-world training on local and swarm models for both external test cohorts (UKA, MHA). We evaluated and compared ResNet101-3D models performance based on AUROC using DeLong's test, considering a significance level of $p<0.05$ and median patient scores from five repetitions for determining better performance. All the p-values below the significance level are highlighted in bold.

Technique	Hyperparameters	Parameters in Million	Model size in mb	Time for 50 epochs with SL model in minutes	Time for 50 epochs with centralized model in minutes
**3D-ResNet18**	Batch size = 1<br>Optimizer = Adam<br>Learning rate = 1e-5<br>(weight decay = 1e-5)<br>Sync interval = 512 batches	33.16	398.08	127	77
**3D-ResNet50**		63.47	761.90	169	122
**3D-ResNet101**		123.65	1484.27	262	150
**3D-DenseNet121**		11.24	135.86	215	142
**ViT-MIL**	Batch size = 32<br>Sync interval = 8 batches	6.57	26.62	33	2.5
**ViT-LSTM-MIL**		31.57	126.67	33	2.6
**Att-MIL**		0.17	1	26	2.1
**2D-ResNet50**	Batch size = 32<br>Sync interval = 512 batches	23.9	98	133	98

Suppl. Table 12: Hyperparameters and technical details for all approaches. We have recorded different Techniques that refer to the method/approach used in the paper. Hyperparameters are details of key hyperparameters for architectural choices. Parameters are the number of model parameters in millions. Model Size is the size of the model in megabytes. Note that the MIL approaches involved an additional feature extraction step, which was not required in the 3D approach. Time for 50 epochs with SL is the duration in minutes for the model to complete 50 epochs using SL. Time for 50 epochs with a centralized model is the duration in minutes for the model to complete 50 epochs using centralized learning.
