# Supplementary material for: Swarm learning with weak supervision enables automatic breast cancer detection in magnetic resonance imaging
Source: Commun Med (Lond). 2025 Feb 6;5:38. doi: 10.1038/s43856-024-00722-5 (PMC11802753; doi:10.1038/s43856-024-00722-5)
Supplement: Supplementary file 3 — Description of Additional Supplementary Files [file 43856_2024_722_MOESM3_ESM.pdf]

Description of Additional Supplementary File

File name: Supplementary Data 1

File description: The data for different image acquisition parameters can be found in 'Supplementary Data 1' (GR = Gradient Echo, SE = Spin Echo, DCE = Dynamic Contrast Enhancement).

File name: Supplementary Data 2

File description: The raw data for prediction performance, measured as AUROC, is provided in 'Supplementary Data 2' for experiments conducted with five repetitions using different nodes and techniques on the internal validation 20% Duke cohort.

File name: Supplementary Data 3

File description: Similarly, the raw data for prediction performance (AUROC) on the external validation UKA cohort, also conducted with five repetitions using different nodes and techniques, is available in 'Supplementary Data 3'.

File name: Supplementary Data 4

File description: The source data for Figure 2 is included in 'Supplementary Data 4'.
