# Supplementary material for: Swarm learning with weak supervision enables automatic breast cancer detection in magnetic resonance imaging
Source: Commun Med (Lond). 2025 Feb 6;5:38. doi: 10.1038/s43856-024-00722-5 (PMC11802753; doi:10.1038/s43856-024-00722-5)
Supplement: Supplementary file 8 — REPORTING SUMMARY [file 43856_2024_722_MOESM8_ESM.pdf]

nature portfolio

Corresponding author(s): Jakob Nikolas Kather

Last updated by author(s): Nov 29, 2024

Reporting Summary

Nature Portfolio wishes to improve the reproducibility of the work that we publish. This form provides structure for consistency and transparency in reporting. For further information on Nature Portfolio policies, see our Editorial Policies and the Editorial Policy Checklist.

Statistics

For all statistical analyses, confirm that the following items are present in the figure legend, table legend, main text, or Methods section.

n/a	Confirmed	
☐	☒	The exact sample size (*n*) for each experimental group/condition, given as a discrete number and unit of measurement
☐	☒	A statement on whether measurements were taken from distinct samples or whether the same sample was measured repeatedly
☐	☒	The statistical test(s) used AND whether they are one- or two-sided<br>*Only common tests should be described solely by name; describe more complex techniques in the Methods section.*
☐	☒	A description of all covariates tested
☐	☒	A description of any assumptions or corrections, such as tests of normality and adjustment for multiple comparisons
☐	☒	A full description of the statistical parameters including central tendency (e.g. means) or other basic estimates (e.g. regression coefficient) AND variation (e.g. standard deviation) or associated estimates of uncertainty (e.g. confidence intervals)
☐	☒	For null hypothesis testing, the test statistic (e.g. *F*, *t*, *r*) with confidence intervals, effect sizes, degrees of freedom and *P* value noted<br>*Give P values as exact values whenever suitable.*
☒	☐	For Bayesian analysis, information on the choice of priors and Markov chain Monte Carlo settings
☐	☒	For hierarchical and complex designs, identification of the appropriate level for tests and full reporting of outcomes
☒	☐	Estimates of effect sizes (e.g. Cohen's *d*, Pearson's *r*), indicating how they were calculated

Our web collection on statistics for biologists contains articles on many of the points above.

Software and code

Policy information about availability of computer code

Data collection	Hospital PACS Systems:<br>Data were retrieved from Picture Archiving and Communication Systems (PACS) at participating institutions. These systems are standard for storing and managing medical imaging data. Specific PACS vendors included:<br>Duke Dataset: General Electric and Siemens PACS systems (version not specified).<br>USZ Dataset: Siemens Syngo PACS (version not specified).<br>CAM Dataset: GE Healthcare PACS (version not specified).<br>UKA Dataset: Philips IntelliSpace PACS (version not specified).<br>MHA Dataset: Siemens Syngo PACS (version not specified).<br>DICOM File Processing:<br>Software: The DICOM data was processed using the following tools:<br>dcm2niix (version 1.0.20220720): Open-source software used for converting DICOM files to NIfTI format.<br>Python (version 3.9): Scripts for data preprocessing and standardization.<br>SimpleITK (version 2.1.1): Open-source library for DICOM file handling and manipulation.
Data analysis	Swarm Learning Framework:<br>Hewlett Packard Enterprise (HPE) Swarm Learning platform (version 2.2.0): Used for implementing swarm learning with decentralized nodes across multiple sites.<br>Custom Python code for swarm model orchestration (available at GitHub Repository).<br>Deep Learning Models:<br>TensorFlow (version 2.10.0) and PyTorch (version 1.13.0): Used for building, training, and validating 2D and 3D convolutional neural network models, including ResNet and DenseNet architectures.

MONAI (version 1.0): Open-source medical imaging deep learning library for preprocessing and model training.
Statistical Analysis and Visualization:
Scikit-learn (version 1.1.3): For calculating AUROC, sensitivity, specificity, F1 score, and other metrics.
Matplotlib (version 3.6.2): For generating visualizations, including performance curves and heatmaps.
DeLong's Test Implementation: Custom Python script based on the roc_analysis module.
Explainability Methods:
GradCAM++: Implemented using PyTorch's Captum library (version 0.6.0).
Occlusion Sensitivity Analysis: Custom Python code leveraging TensorFlow and PyTorch libraries.

For manuscripts utilizing custom algorithms or software that are central to the research but not yet described in published literature, software must be made available to editors and reviewers. We strongly encourage code deposition in a community repository (e.g. GitHub). See the Nature Portfolio guidelines for submitting code & software for further information.

Data

Policy information about availability of data

All manuscripts must include a data availability statement. This statement should provide the following information, where applicable:

- Accession codes, unique identifiers, or web links for publicly available datasets
- A description of any restrictions on data availability
- For clinical datasets or third party data, please ensure that the statement adheres to our policy

The Duke dataset analyzed in this study can be accessed from The Cancer Imaging Archive by visiting https://doi.org/10.7937/TCIA.e3sv-re93. All other datasets analyzed during the current study are available from the authors at the respective site upon reasonable request. This entails setting up a collaboration agreement between the participating institutions and is contingent on the local ethics board agreeing to data sharing. Any requests should be directed at all authors from the respective centers and will be responded to within four weeks.

Human research participants

Policy information about studies involving human research participants and Sex and Gender in Research.

Reporting on sex and gender	NA
Population characteristics	NA
Recruitment	NA
Ethics oversight	NA

Note that full information on the approval of the study protocol must also be provided in the manuscript.

Field-specific reporting

Please select the one below that is the best fit for your research. If you are not sure, read the appropriate sections before making your selection.

☒ Life sciences ☐ Behavioural & social sciences ☐ Ecological, evolutionary & environmental sciences

For a reference copy of the document with all sections, see nature.com/documents/nr-reporting-summary-flat.pdf

Life sciences study design

All studies must disclose on these points even when the disclosure is negative.

Sample size	In this study, five breast MRI datasets were utilized, comprising both training and external test cohorts. The training cohorts included Duke (651 patients analyzed after excluding 271 cases lacking tumor location), USZ (272 patients), and CAM (302 patients out of 305 collected). For external testing, UKA (422 patients analyzed from 500 collected) and MHA (144 patients analyzed from 145 collected) were used. This comprehensive dataset included diverse populations to support robust analysis and validation.
Data exclusions	The data was excluded based on the target for prediction. The exclusion reason and count is clearly explained in the CONSORT chart.
Replication	The measures are taken to replicate are reproduce of all the experiments are the hyper parameters and kept constant and are saved and documented. The ID of patient are stored during different splits for training at different internals. The model parameters are documented and the models are saved.
Randomization	The random selection of patients to show data efficiency of swarm learning is made possible by proving a random seed and dividing the patients. For reputation of experiments the random seed is documented.
Blinding	The clinical data of available during the analysis so the there was no need for any blinding in our study.

Reporting for specific materials, systems and methods

We require information from authors about some types of materials, experimental systems and methods used in many studies. Here, indicate whether each material, system or method listed is relevant to your study. If you are not sure if a list item applies to your research, read the appropriate section before selecting a response.

Materials & experimental systems

n/a	Involved in the study	
☒	☐	Antibodies
☒	☐	Eukaryotic cell lines
☒	☐	Palaeontology and archaeology
☒	☐	Animals and other organisms
☐	☒	Clinical data
☒	☐	Dual use research of concern

Methods

n/a	Involved in the study	
☒	☐	ChIP-seq
☒	☐	Flow cytometry
☒	☐	MRI-based neuroimaging

Clinical data

Policy information about clinical studies

All manuscripts should comply with the ICMJE guidelines for publication of clinical research and a completed CONSORT checklist must be included with all submissions.

Clinical trial registration	*Provide the trial registration number from ClinicalTrials.gov or an equivalent agency.*
Study protocol	*Note where the full trial protocol can be accessed OR if not available, explain why.*
Data collection	*Describe the settings and locales of data collection, noting the time periods of recruitment and data collection.*
Outcomes	*Describe how you pre-defined primary and secondary outcome measures and how you assessed these measures.*
